# Myelodysplastic Syndrome in a Patient With Common Variable Immunodeficiency: A Rare Occurrence

**DOI:** 10.7759/cureus.28690

**Published:** 2022-09-02

**Authors:** Nihas R Mateti, Raju K Vaddepally, Priya Elsa Skaria, Abhinav B Chandra

**Affiliations:** 1 Department of Medicine, Osmania Medical College, Hyderabad, IND; 2 Oncology, Yuma Regional Medical Center, Yuma, USA; 3 Hematopathology, Yuma Regional Medical Center, Yuma, USA

**Keywords:** hypercellular bone marrow, hemato-oncology, myelodysplastic (mds)/myeloproliferative neoplasm (mpn) disease spectrum, common variable immunodeficiency (cvid), cvid and mds

## Abstract

Common variable immunodeficiency (CVID) is a primary immunodeficiency disorder caused by impaired B-cell function and antibody production. It commonly presents with chronic sinopulmonary and gastrointestinal manifestations. It is also associated with transformation to acute myeloid leukemia. However, the association of CVID with myelodysplastic syndrome (MDS) is rare. This case report aims to present one such rare association in a 26-year-old patient presenting with severe thrombocytopenia. Bone marrow biopsy revealed hypercellular marrow with 80-90% cellularity along with an increase in CD34 blasts. Cytogenetics revealed loss of the Y chromosome. Diagnosis of MDS with excess blasts-2 was confirmed with a Revised International Prognostic Scoring System score of 4, placing the patient in the intermediate-risk category. The patient was started on azacitidine, a hypomethylating agent. A referral to a bone marrow transplant was also done for the consideration of an allogeneic stem cell transplant.

## Introduction

Common variable immunodeficiency (CVID) is a primary immunodeficiency disorder caused by impaired B-cell function and antibody production. Although patients present with varied clinical presentations, the presence of chronic sinopulmonary and gastrointestinal manifestations are characteristic manifestations. It is also associated with autoimmune disorders (immune thrombocytopenic purpura (ITP) and autoimmune hemolytic anemia (AIHA)), inflammatory conditions (lymphoid hyperplasia, granulomatous infiltrations, and inflammatory bowel disease), and malignancies (non-Hodgkin lymphoma, leukemia, and gastric carcinoma) [1-3]. An extensive literature review for myelodysplastic syndrome (MDS) incidence in CVID using the keywords CVID+MDS, myelodysplastic syndrome in CVID in PubMed, and Google Scholar revealed it to be a rare occurrence with only a few reported cases [3,4]. Here, we report one such rare incidence of MDS in a patient with CVID.

## Case presentation

A 26-year-old with a history of CVID since childhood, on treatment with intravenous (IV) immunoglobulin every three weeks, presented to a hematologist, with an abnormal blood picture (Table 1).

**Table 1:** Abnormal investigations compared to the patient's previous visit nine years ago

	At the time of presentation	Nine years ago	Reference range
Hemoglobin (g/dL)	11.1 (↓)	15.5	13–17
Platelet count (×10³ cells/μL)	19 (↓↓)	53 (↓)	150–400
White blood cell count (×10³ cells/μL)	3.9 (↓)	3.2 (↓)	4.0–10.0
Absolute neutrophil count (cells/μL)	2,000 (↓)	1,280 (↓↓)	2,500–6,000
Immunoglobulin (Ig) levels (mg/100 mL)	-	IgA: 97, IgG: 826, IgM: 118	IgA: 81–211, IgG: 688–1,251, IgM: 65–132

The patient’s platelet count had remained stable between 50,000 and 75,000 cells/μL between appointments. The patient also complained of 40 to 50-pound intentional weight loss.

**Figure 1:**
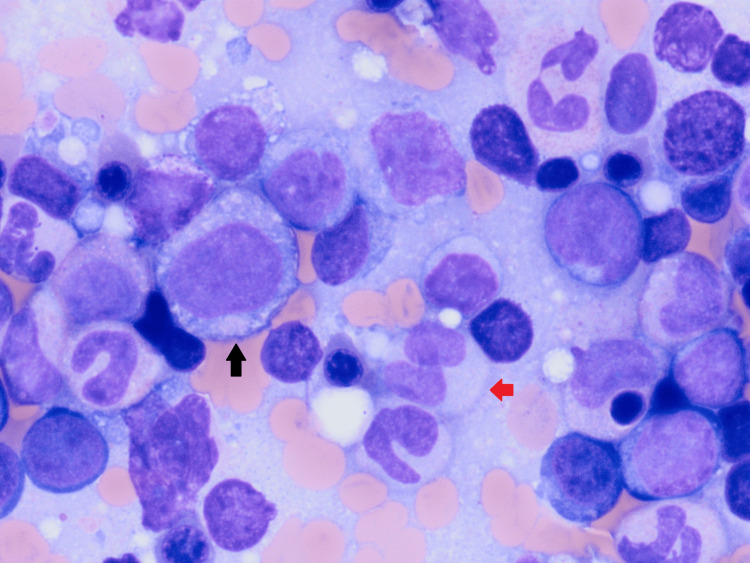
Diagnostic bone marrow aspirate with evidence of myelodysplasia (100× oil objective or 1,000× magnification). Dysplastic immature myeloid precursors (black arrow) and mature neutrophils with abnormal and decreased cytoplasmic granulation (red arrow).

**Figure 2:**
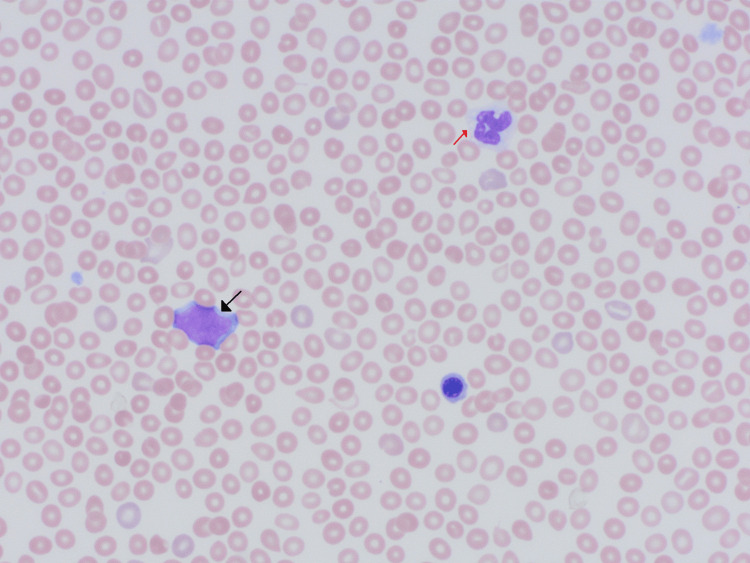
Concurrent peripheral blood smear with leukoerythroblastosis and myeloid dysplasia (50× oil objective or 500× magnification). The image displays a circulating blast with fine nuclear chromatin and scant pale blue cytoplasm (black arrow) and dysplastic mature neutrophils with decreased to absent cytoplasmic granulation (red arrow).

A bone marrow biopsy showed hypercellular bone marrow with 80-90% cellularity. An increase in CD34-positive blasts of about 15-16% of total cells was noted (Figures 1, 2). Cytogenetics revealed abnormal male karyotype-45, X,-Y(20), and loss of the Y chromosome. *FMS-like tyrosine kinase-3* (*FLT-3*) mutation, commonly associated with acute myeloid leukemia (AML), was negative. Normal results were noted in chromosomes 5, 7, 8, 11, and 20 probe sets. A diagnosis of MDS with excessive blasts (MDS-EB) was made. No abnormalities were detected in the following genes: *FLT3*, *IDH1*, *IDH2*, and *NPM*. Next-generation sequencing revealed the following variants: *IKZF1 N159S*; *KIT D816V*; *KRAS K117N*; NF1 R711Pfs*37; *NRAS G60V, G12D*; and *PHF6 R225*.

A computed tomography scan of the chest, abdomen, and pelvis revealed the progression of splenomegaly to 26 cm (compared to 19 cm in 2018). Thrombocytopenia was autoimmune, due to CVID, with further worsening secondary to MDS-EB. Our patient had a loss of chromosome Y, which is a very good prognostic marker. A revised International Prognostic Scoring System (IPSS-R) score of 4 placed the patient in the intermediate-risk category. IPSS-RA score of 2.68 places a patient in the low-risk category. According to the National Comprehensive Cancer Network (NCCN) guidelines, the patient was started on a hypomethylating agent, azacitidine. A referral to a bone marrow transplant was also done for the consideration of an allogeneic stem cell transplant. The patient passed away three months after initiation of the treatment due to infective endocarditis.

## Discussion

The association of MDS with CVID is quite unusual. CVID disorders are rare autosomal recessive disorders with a mean incidence of 0.676 ± 0.83 per 100,000 globally, with incidence increasing to 3.374 per 100,000 in developed nations [5]. Although most cases are diagnosed between the ages of 20 and 40, there are cases at either end of the spectrum [6]. Common mutations associated with CVID disorders are *TACI*, *BAFF-R*, *CD19*, *CD81*, *CD20*, *CD21*, *TWEAK*, and *NFKB2* [7]. Kiaee et al., in a systemic analysis, revealed the prevalence of malignancy in CVID at 8.6% (95% confidence interval (CI) = 7.1-10.0; I^2^ = 79.2%) [8]. In a meta-analysis, Mayor et al. showed that men in the CVID subgroup were diagnosed with a significantly higher rate of cancer than the age-adjusted male population (n = 48 vs. n = 27.5; p < 0.001), while women with CVID were diagnosed with cancer at a similar rate as the age-adjusted female population (n = 71 vs. n = 64.3, p = NS) [3]. Splenomegaly is a common manifestation present in 30% of patients with CVID [9]. The prevalence of lymphoma, gastric cancer, and breast cancer was 4.1%, 1.5%, and 1.3%, respectively [8]. There have been only a few reported cases of MDS associated with CVID.

MDS is a hematological malignancy with ineffective hematopoiesis due to de novo mutation primarily in oncogenic genes. It most commonly occurs in individuals greater than 65 years of age, with a mean age of 77 and a male preponderance [10]. Patients are usually asymptomatic but sometimes present with bone marrow failure features such as fatigue, infections, and ecchymosis. It is usually associated with transformation to AML in about 9.6% of patients, increasing to 24.6% among transfused [10]. Common mutations associated with MDS disorders are *SF3B1*, *TET2*, *SRSF2*, *ASXL1*, *DNMT3A*, *RUNX1*, *U2AF1*, *TP53*, and *EZH2 *[11]. MDS is classified into various subtypes based on peripheral blood smear and bone marrow findings (Table 2).

**Table 2:** MDS classification according to the WHO. *Cytopenia: anemia (hemoglobin <10 g/dL) or thrombocytopenia (platelet count <100 × 10^3^cells/μL) or absolute eosinophil count <1,800 cells/μL. MDS: myelodysplastic syndrome; WHO: World Health Organization Hong M, He G. The 2016 revision to the World Health Organization classification of myelodysplastic syndromes. J Transl Int Med. 2017, 5:139-43 [12].

Subtype	Peripheral blood smear findings	Bone marrow findings
MDS with ring sideroblasts (MDS-RS)	Anemia with no blasts	Dysplasia in any one hematopoietic lineage, blasts <5%, erythroid precursors with ring sideroblasts ≥15%
MDS with single lineage dysplasia (MDS-SLD)	One to two cytopenias, blast cells without Auer rods <1%	Dysplasia in any one hematopoietic lineage, blast cells without Auer rods <5%, ring sideroblasts <15%
MDS with multi-lineage dysplasia (MDS-MLD)	One to three cytopenias, blast cells without Auer rods <1%	Dysplasia in ≥2 hematopoietic lineage, blast cells without Auer rods <5%, ring sideroblasts ~15%
MDS with isolated del(5q)	One to two cytopenias - most common are severe anemia and thrombocytopenia	Erythroid dysplasia, blast cells without Auer rods <5%
MDS with excess blasts-1 (MDS-EB-1)	One to three cytopenias, blast cells without Auer rods <2–4%	Dysplasia in 1–3 hematopoietic lineage, blast cells without Auer rods 5–9%, no ring sideroblasts
MDS with excess blasts-2 (MDS-EB-2)	One to three cytopenias, blast cells <5–19%, Auer rods may be present	Dysplasia in 1–3 hematopoietic lineage, blast cells 10–19%, Auer rods may be present
MDS, unclassifiable (MDS-U)	≥1 cytopenia, ≥1% blasts on two different occasions	Unilineage dysplasia or no dysplasia, blast cells without Auer rods <5%, characteristic MDS cytogenetics

Investigations for the diagnosis of MDS include peripheral blood studies (routine blood counts, differential blood counts, lactate dehydrogenase, ferritin, human leukocyte antigen typing) and bone marrow studies (morphology, karyotype, cytogenetics, immunophenotyping, histological analysis, mutation analysis) [13]. Previously reported cases of MDS in CVID had diagnoses at 78 and 75 years of age, which is the common age of presentation for MDS [4]. In our case, MDS was diagnosed at a very young age ruling out an incidental occurrence. Common gene mutations associated with MDS such as *ASXL1 *and *RUNX1*, which were positive in previously reported cases, were negative in our case. However, *KRAS *mutation, which was positive in our case, has been reported in association with MDS, suggesting a possible underlying pathological mechanism [14]. GATA2 deficiency can be considered as a possible underlying mechanism for MDS in hypogammaglobulinemia; however, the presence of hypercellular marrow rules out GATA2 deficiency in our case.

Treatment options range from a regular follow-up to low-intensity therapy (hypomethylating agents, biological response modifiers, and immunosuppressive therapy) to high-intensity therapy (induction chemotherapy or hematopoietic stem cell transplantation (HSCT)) [15]. Treatment is chosen based on risk stratification, prognostic outcomes, and the risk of leukemic transformation. Prognostic outcomes can be calculated using IPSS-R. The NCCN guidelines for low-risk patients (IPSS: low/intermediate-1; IPSS-R: very low, low, intermediate) with 5q deletion can be treated using lenalidomide [16]. Those with epoetin alfa >500 with a good probability of response to immunosuppressive therapy (IST) can be treated with antithymocyte globulin and cyclosporine A. Those with a poor probability of response to IST can be treated with Azacitidine or Decitabine. High-risk patients (IPSS: intermediate-2; high IPSS-R: intermediate, high, very high; WPSS: high, very high) who are transplant candidates should undergo HSCT. Those who cannot undergo transplants can be treated with hypomethylating agents.

## Conclusions

CVID is a rare primary immunodeficiency disorder caused by impaired B-cell function. Due to its more common association with autoimmune disorders (ITP and AIHA) and hematological malignancies (leukemia and non-Hodgkin lymphoma), abnormalities in common blood reports such as cytopenias have to be thoroughly investigated. With growing evidence for the presence of MDS in CVID patients, physicians should have a low threshold for suspicion of MDS. Peripheral blood smear and bone marrow aspirate findings help establish and categorize MDS. The cytogenetic analysis combined with molecular genetic testing aids in defining prognosis and treatment decision-making. Further studies are needed to find specific markers to identify MDS in CVID patients.
